# Case Report: Penile hamartoma with penile torsion in a child: etiology-targeted one-stage surgical correction and literature review

**DOI:** 10.3389/fped.2025.1715759

**Published:** 2025-12-11

**Authors:** Zhigang Yao, Chenghao Zhanghuang, Nian Zhou, Jinrong Li, Zipeng Hao, Bing Yan, Hui Zhao

**Affiliations:** 1 Department of Urology, Kunming Children’s Hospital (Children’s Hospital Affiliated to Kunming Medical University), Kunming, China; 2 Urology Department, The First Affiliated Hospital of Kunming Medical University, Kunming, China; 3 Yunnan Province Clinical Research Center for Children’s Health and Disease, Yunnan Key Laboratory of Children’s Major Disease Research, Kunming, China; 4 Department of Dermatology, Kunming Children’s Hospital (Children’s Hospital Affiliated to Kunming Medical University), Kunming, China

**Keywords:** penis, hamartoma, pediatric, penile torsion, surgery

## Abstract

**Background:**

Pediatric penile hamartoma is extremely rare. Preoperative imaging often cannot definitively characterize the lesion, and histopathology remains the diagnostic gold standard. We report a child with penile hamartoma and torsion, discuss management, and compare outcomes with the literature.

**Methods:**

We retrospectively analyzed the clinical presentation, imaging, intraoperative findings, and pathology. Relevant reports were reviewed for comparison.

**Results:**

Complete excision of a ventral hamartomatous appendage plus circumcision and release of a fibrous tethering band achieved immediate torsion correction in a single stage. Histopathology showed stratified squamous epithelium with proliferative fibrous and adipose tissue containing nerve bundles, ganglion cells, and focal smooth muscle—consistent with hamartoma. Recovery was uneventful; at 12 months no recurrence was observed.

**Conclusion:**

Etiology-targeted, one-stage correction—degloving (circumcision), release of tethering bands, complete lesion excision, and simultaneous torsion repair—can be safe and effective. Long-term follow-up is advised.

## Introduction

A hamartoma is a tumor-like malformation formed by disorganized overgrowth of tissue native to the involved site during development. Penile hamartomas are exceedingly rare in children and may mimic other tumors or genital anomalies (1–3). Imaging delineates extent and relationships, whereas histopathology confirms diagnosis. We describe a pediatric penile hamartoma with significant torsion and highlight an etiology-targeted, one-stage strategy.

## Case report

### Presentation and examination

**Figure 1:**
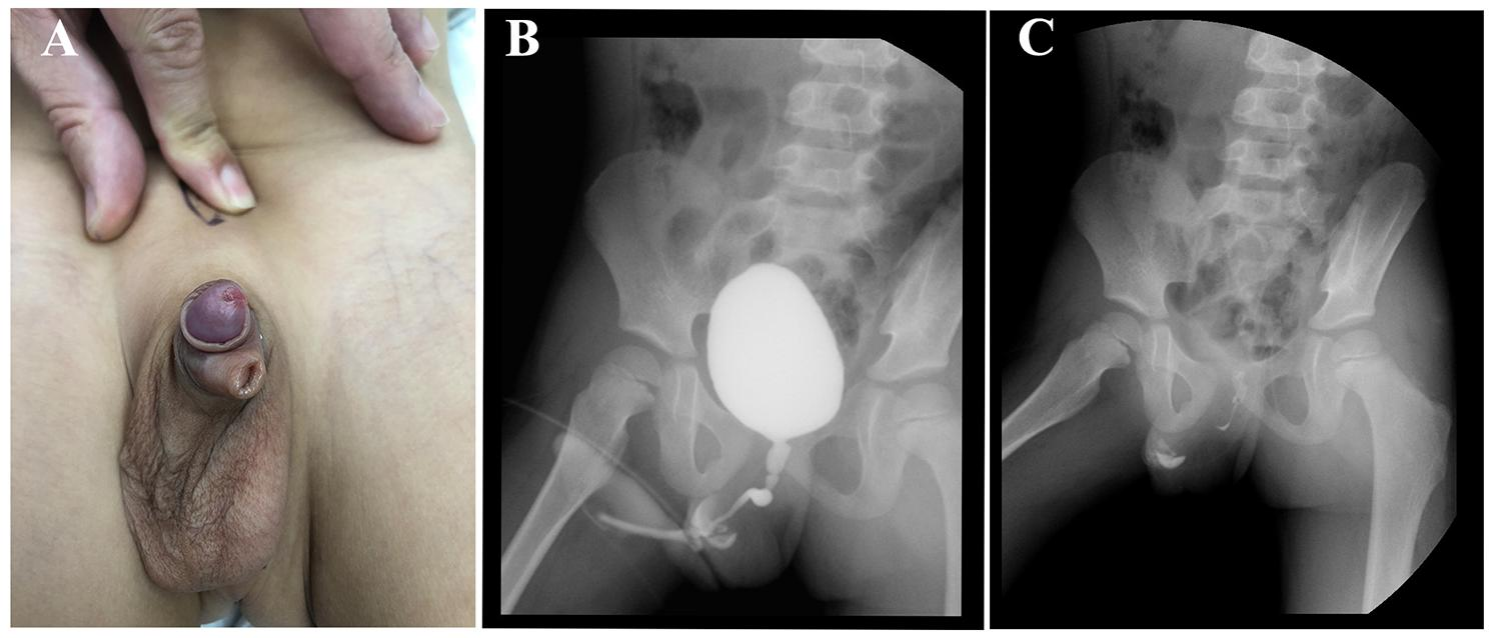
Preoperative clinical and imaging evaluation. **(A)** Clinical photograph showing a ventral, skin-covered appendage and approximately 90° counterclockwise penile torsion with an eccentric meatus. **(B)** Voiding cystourethrogram (VCUG), filling/early voiding phase: normal bladder contour with opacification and a single urethral channel; no duplicated tract is seen. **(C)** VCUG, post-void/late-phase plain film: no secondary urethral tract or fistulous communication is demonstrated.

A 4-year-3-month-old boy was brought to our clinic with an abnormal penile appearance noted since early infancy (approximately 4 years in duration). The child's overall growth and development were normal. On genital examination, the penis appeared to have an excessive foreskin and was rotated about 90° counterclockwise, such that the urethral meatus was displaced laterally. An 2 cm skin-covered appendage was evident on the ventral side of the penile shaft; it was soft, lacked any palpable corpus cavernosum-like structure, and was covered with skin resembling the prepuce. Both testes were palpated within the scrotum with normal consistency, and the scrotal appearance was unremarkable (Figure 1A).

### Investigations

The mother's prenatal exams had been normal. The patient had no history of surgery, trauma, or a family history of tumors. Laboratory tests, including serum alpha-fetoprotein (AFP), carcinoembryonic antigen (CEA), and human chorionic gonadotropin (β-HCG), were all within normal ranges. Routine preoperative evaluations (chest x-ray, electrocardiogram, and cardiac ultrasound) showed no abnormalities. An excretory urography study (intravenous urogram) revealed no obvious abnormality of the bladder; both ureters were not visualized, and after removing the catheter, the patient voided normally with no evidence of a duplicated urethra on the study (Figures 1B,C). Based on the clinical and imaging findings, the provisional diagnosis was “penile torsion with penile anomaly of unclear nature (to be determined)”. No contraindications for surgery were present, so elective surgical treatment was planned.

### Surgery and intraoperative findings

**Figure 2:**
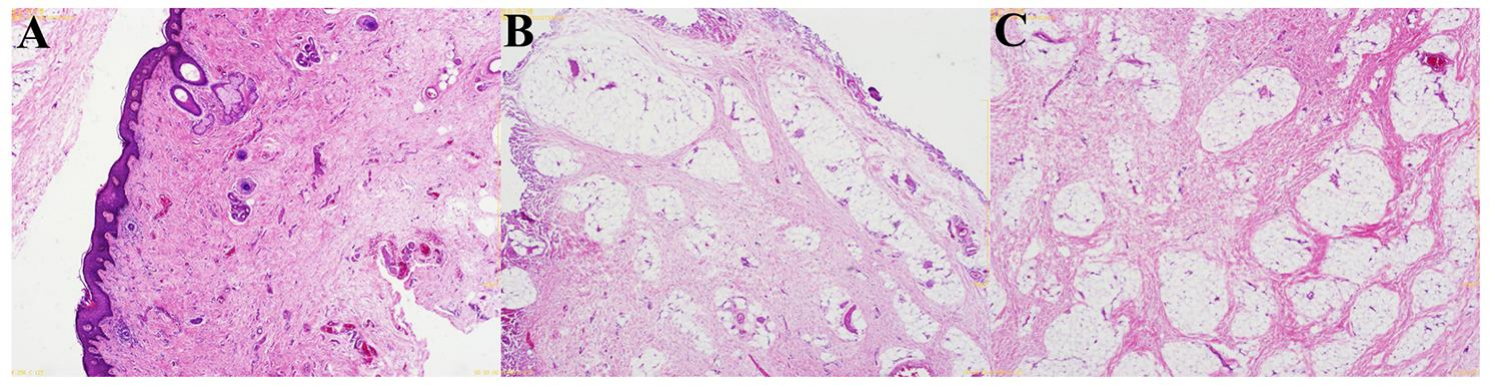
Intraoperative course and one-stage correction. **(A)** After degloving (circumcision), persistent axial torsion is evident with the catheter *in situ* and a ventral appendage. **(B)** Dissection identifies a cord-like fibrous tether between the appendage and the ventral shaft/corpus spongiosum; the traction band is released and excised *en bloc*. **(C)** Final appearance after complete excision and torsion repair, showing restored alignment and a well-healed circumferential suture line.

Under general anesthesia, a circumferential incision was made at the tip of the ventral appendage and dissection was carried proximally. The ventral penile appendage was found to consist of a cord-like fibrous tissue that was attached to the tunica of the corpus spongiosum; it was well demarcated from surrounding structures. The cord-like band was the apparent source of traction causing the penile torsion. The fibrous band and attached appendage were completely excised *en bloc* and sent for pathological examination (Figures 2A,B). Next, a circumferential degloving incision (circumcision) was made approximately 0.5 cm below the coronal sulcus, and the abnormally tethered fibrous bands in the subcutaneous tissue were carefully released. Once these traction bands were freed, the penis could be rotated back to its normal alignment and straightened without tension, and the urethra was confirmed to be intact and of normal length. A urethral catheter was left in place postoperatively and removed on postoperative day 3, after which the child voided without difficulty (Figure 2C). The surgical correction was completed in a single stage with an excellent immediate result.

### Pathology and follow-up

**Figure 3:**
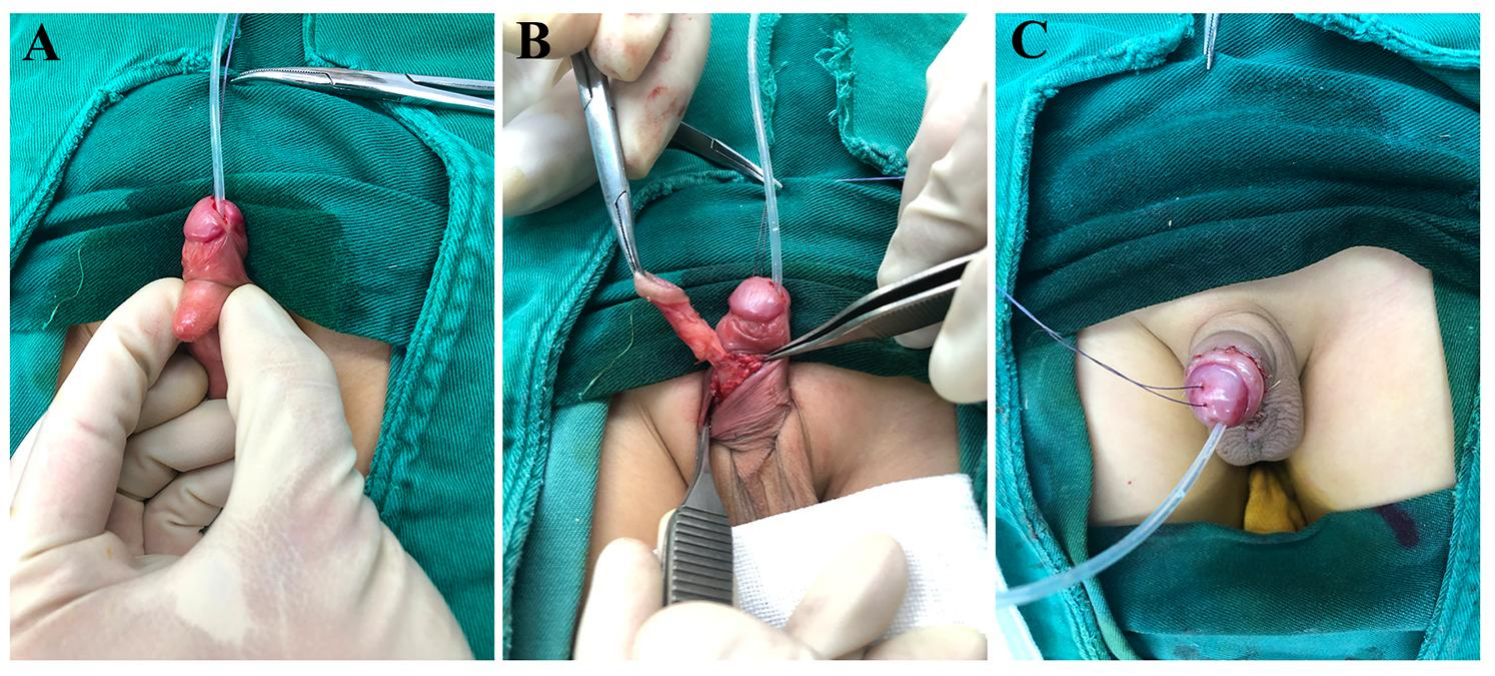
Histopathology of the penile hamartoma (hematoxylin–eosin, HE). **(A)** Low-power view (original magnification ×40) showing a surface of stratified squamous epithelium with an underlying disorganized overgrowth of fibrous and adipose tissue. **(B)** Intermediate-power view (original magnification ×100) highlighting scattered nerve bundles (NB) and ganglion cells (GC) within the lesion, together with small blood vessels. **(C)** High-power view (original magnification ×200) demonstrating focal bundles of smooth muscle (SM) embedded in the fibrous stroma, a composition consistent with a hamartomatous lesion of the penis.

Gross examination of the excised tissue confirmed a fibrous cord tethering the ventral appendage to the penile shaft. Histopathological analysis showed that the lesion was covered by stratified squamous epithelium. Beneath the epithelium, there was an overgrowth of fibrous and adipose tissue, within which nerve bundles, ganglion cells, and blood vessels were observed; focal small bundles of smooth muscle were also present (Figures 3A–C). These features were consistent with a hamartoma of the penis. The postoperative diagnosis was finalized as “penile torsion secondary to penile hamartoma.” The patient had an uneventful recovery and was discharged on postoperative day 3. At the 1-year follow-up visit, no recurrence or complications had occurred. The penis appeared normal in shape, and urinary function (voiding stream and continence) was excellent.

## Discussion

Penile hamartomas in pediatric patients are exceptionally rare. Clinically, they usually present as an abnormal penile appearance or a localized penile mass noticed by caregivers. While imaging studies can aid in assessing the extent of the lesion and its relationship to adjacent structures, the definitive diagnosis depends on pathological examination of the excised tissue (4, 5). In our case, the pathology demonstrated the characteristic disorganized proliferation of tissues native to the penis (fibrous, adipose, nerve, vascular, and smooth muscle elements) beneath a squamous epithelial surface, and the intraoperative findings confirmed a fibrous cord-like connection between the hamartomatous appendage and the corpus spongiosum.

We conducted a literature search of PubMed, PMC and major journal databases up to September 18, 2025. Keywords used included combinations of terms such as “penile hamartoma”, “hamartomatous lesion”, “dermoid cyst or epidermoid cyst of the penis/scrotum”, and “fibroepithelial polyp” coupled with “child”, “infant”, or “pediatric”. Relevant articles’ references were also reviewed (snowball method). Our review focused on true hamartomas and hamartomatous lesions occurring in the penile proper (glans, shaft, base, or prepuce/penile skin) in patients under 18 years of age. This search yielded only three prior reported cases that fit these criteria (6–8), which are summarized in Table 1.

**Table 1:** Comparison of the present case with previously reported pediatric penile hamartomatous lesions.

Case	First author (Year)	Lesion location/histology (diagnosis)	Age	Penile torsion?	Traction band noted?	One-stage correction?	Main surgical procedure	Follow-up (months)	Recurrence
Present case	— (2025)	Ventral penile hamartoma (fibrous/fatty/nerve/vascular tissue ± smooth muscle foci)	4 years	Yes	Yes	Yes	Degloving + release of tethering band + complete excision	12	No
1	Patel (2014) (5)	Glans dermoid cyst (true hamartoma)	Preschool (exact age NR)	No	No	Yes	Cyst excision ± circumcision	NR	No
2	Yildirim (2004) (6)	Glans fibroepithelial polyp (hamartomatous proliferation)	4 years	No	No	Yes	Polyp excision + simultaneous circumcision	NR	No
3	Şencan (2015) (7)	Glans fibroepithelial polyp (hamartomatous proliferation)	6 months	No	No	Yes	Complete excision + ruled out urethral communication	12	No

NR, not reported.

Compared to the previously reported cases, our case has several unique features and clinical implications. First, an etiological clue for the torsion was clearly identified. In prior reports, penile or peri-urethral hamartomas were generally described as “incidental masses”, and a direct cause-and-effect relationship between a tethering band and penile torsion was not well established (6). In our case, a distinct cord-like hamartomatous tissue was found to be the source of a significant 90° penile torsion. Releasing this tether and excising the lesion led to immediate torsional correction, suggesting that an etiology-targeted surgical approach (i.e., removing the traction source) is superior to simple excision of the mass alone when torsion is present. Second, our management strategy was more integrated. Most previously reported cases were managed with either staged procedures or treatment of only the localized lesion (7). In contrast, our case was managed in a one-stage operation that combined complete lesion excision, foreskin degloving/circumcision, and torsion repair concurrently. This one-stop approach achieved an optimal cosmetic and functional outcome, and no recurrence was observed at one year postoperatively. Third, our diagnostic workup provides a more replicable pathway for differentiation from other conditions. Preoperatively, we performed an excretory urography study to exclude urethral duplication (no double urethra was visualized), and we assessed whether any corpus cavernosum/urethra-like structures were present in the appendage as well as the anatomical axis deviation (2, 4, 8). These steps helped avoid misdiagnosis with conditions such as fibrous hamartoma of infancy (a benign fibrous tumor in infants, FHI), fibrolipoma, teratoma or dermoid cyst, vascular malformations, or pseudodiphallia (accessory pseudo-penis). This systematic approach reduced the risk of misidentifying the lesion, ensuring appropriate management.

In terms of surgical correction techniques for torsion, mild to moderate isolated penile torsion can often be corrected by simply degloving (circumferential skin mobilization) and realigning the skin to the proper position. However, in more severe torsion cases (>90°) or those with deeper fascial abnormalities or a tethering band, some authors have recommended additional maneuvers such as dorsal tunica albuginea plication or rotational skin/dartos flaps to prevent residual torsion (9). In our case, by precisely identifying and removing the fibrous traction band, we achieved full correction of a ∼90° torsion without the need for any dorsal plication or flap, illustrating that in a “traction-induced torsion” scenario, a simplified surgery addressing the root cause can be effective. For postoperative evaluation, we advocate documenting objective cosmetic outcomes, parental satisfaction, and voiding function parameters, which would allow comparison of results across cases and provide evidence for long-term success (10, 11).

Additionally, this case highlights the importance of a systematic risk assessment for underlying syndromes or multiple lesions. If a patient presents with multiple hamartomas, mucocutaneous abnormalities, or other features such as macrocephaly, one should consider the possibility of PTEN hamartoma tumor syndrome (PHTS) and pursue appropriate genetic counseling and surveillance (12–14). In cases where imaging suggests deep tissue involvement or proximity to the urethra, a preoperative urethrogram or magnetic resonance urography (MRU) can be helpful to plan resection margins and reconstructive strategy. Careful preoperative planning in such complex cases can help prevent intraoperative surprises and guide safe excision while preserving function (1).

## Conclusion

In conclusion, pediatric penile hamartomas are extraordinarily rare. Preoperative imaging can be valuable for delineating the lesion's extent and its relationships with surrounding structures, but a definitive diagnosis relies on histopathology. Our case demonstrates that an etiology-targeted, one-stage surgical approach — consisting of complete lesion excision, foreskin degloving (circumcision) with exposure, release of any tethering fibrous bands, and simultaneous correction of penile torsion — can achieve a safe and effective outcome. In this patient, the strategy resulted in excellent cosmetic and functional results with no recurrence during follow-up. Children presenting with multiple hamartomatous lesions or other concerning clinical features should undergo genetic evaluation (for conditions such as PHTS) and warrant long-term surveillance. Moving forward, accumulation of more cases through multi-center collaboration, along with standardized surgical techniques and quantitative outcome measures, is needed to establish stronger evidence and guidance for managing this rare condition.

## Data Availability

The original contributions presented in the study are included in the article/Supplementary Material, further inquiries can be directed to the corresponding author.
